# Signal transducer and activator of transcription-3 licenses Toll-like receptor 4-dependent interleukin (IL)-6 and IL-8 production via IL-6 receptor-positive feedback in endometrial cells

**DOI:** 10.1038/mi.2015.131

**Published:** 2016-01-27

**Authors:** J G Cronin, V Kanamarlapudi, C A Thornton, I M Sheldon

**Affiliations:** 1 Institute of Life Science, College of Medicine, Swansea University, Swansea, UK

## Abstract

Interleukin 6 (IL-6), acting via the IL-6 receptor (IL6R) and signal transducer and activator of transcription-3 (STAT3), limits neutrophil recruitment once bacterial infections are resolved. Bovine endometritis is an exemplar mucosal disease, characterized by sustained neutrophil infiltration and elevated IL-6 and IL-8, a neutrophil chemoattractant, following postpartum Gram-negative bacterial infection. The present study examined the impact of the IL6R/STAT3 signaling pathway on IL-8 production by primary endometrial cells in response to short- or long-term exposure to lipopolysaccharide (LPS) from Gram-negative bacteria. Tyrosine phosphorylation of STAT3 is required for DNA binding and expression of specific targets genes. Immunoblotting indicated constitutive tyrosine phosphorylation of STAT3 in endometrial cells was impeded by acute exposure to LPS. After 24 h exposure to LPS, STAT3 returned to a tyrosine phosphorylated state, indicating cross-talk between the Toll-like receptor 4 (TLR4) and the IL6R/STAT3 signaling pathways. This was confirmed by short interfering RNA targeting the *IL6R*, which abrogated the accumulation of IL-6 and IL-8, induced by LPS. Furthermore, there was a differential endometrial cell response, as the accumulation of IL-6 and IL-8 was dependent on STAT3, suppressor of cytokine signaling 3, and Src kinase signaling in stromal cells, but not epithelial cells. In conclusion, positive feedback through the IL6R amplifies LPS-induced IL-6 and IL-8 production in the endometrium. These findings provide a mechanistic insight into how elevated IL-6 concentrations in the postpartum endometrium during bacterial infection leads to marked and sustained neutrophil infiltration.

## Introduction

The mechanisms of response to pathogens and their pathogen-associated molecular patterns, such as *Escherichia coli* and lipopolysaccharide (LPS), is orchestrated by pattern-recognition receptors, including the Toll-like receptors (TLRs), and the subsequent downstream activation of nuclear factor-κB (NF-κB) and mitogen-activated protein kinase (MAPK) signaling pathways, resulting in the production of inflammatory cytokines.^1, 2^ The signal transducer and activator of transcription 3 (STAT3) pathway orchestrates the inflammatory response through cross-talk with pattern-recognition receptor pathways, such as the TLR family, inducing the production of proinflammatory signaling cytokines, including interleukin (IL)-6.^3, 4^ The multifunctional cytokine IL-6 is produced by many cells, including endometrial cells, in response to infection and damage, and is critical for the pattern of leukocyte recruitment and tissue homeostasis.^1, 2, 5, 6, 7^ During this process, IL-6 signals and activates STAT3 via the cognate IL-6 receptor (IL6R) complex, which consists of a heterodimer of IL6Rα and gp130. Upon ligand binding, the gp130 receptor-associated Janus kinases JAK1, JAK2, and Tyk2 become activated.^8^ The JAKs in turn phosphorylate tyrosine motifs within the cytoplasmic region of gp130 resulting in the association of Src homology domains containing tyrosine phosphatase-2 and activation of the Ras/Raf/MAPK pathway. Activation of JAKs also results in the recruitment of signaling molecules, including STAT3 and suppressor of cytokine signaling 3 (SOCS3), an inhibitor of STAT3.^9^ However, SOCS3 does not directly inhibit STAT3 but acts in a receptor-specific manner, through interference between gp130 and JAK activation.^10, 11^ Alternatively, SOCS proteins can be rapidly induced by pathogen-associated molecular patterns, act as regulators of LPS-induced activation in macrophages, and interact with NF-κB and TLR pathway components, including the adaptor Mal.^12, 13, 14, 15^ Furthermore, SOCS proteins activate MAPK pathways, particularly extracellular signal-regulated kinases (ERK1/2), which is required for endometrial decidualization in mice and humans, and for conception in cows.^16, 17, 18^

During acute inflammation, the chemokine IL-8 initially recruits neutrophils, which are later replaced by a more sustained population of mononuclear cells. IL-6 and its soluble receptor are important for this transition of leukocyte recruitment, but in some diseases the transition fails, demonstrated by persistent neutrophil infiltration.^5, 6^ An exemplar mucosal disease, where persistent neutrophil recruitment is a key feature, is postpartum endometritis in *Bos taurus*. Uterine disease of dairy cows is of major economic importance, costing the European Union dairy industry €1.4 billion per year. Approximately 90% of dairy cattle acquire bacterial contamination of the uterine lumen after parturition, leading to chronic inflammation and endometritis in 40% of cases. Endometritis is refractory to current treatments and leads to significant morbidity, mortality, and infertility.^19^ Persistent inflammation, even following bacterial clearance, is a major feature of the disease and is associated with marked and sustained neutrophil infiltration, elevated IL-6, and IL-8, and pus accumulating in the uterine lumen.^19^ However, the mechanism that drives these events is unclear. We hypothesized that there is cross-talk between TLR signaling and STAT3 pathways in bovine endometrial cells, sustaining the production of IL-6 and IL-8 via IL6R signaling.

## Results

### Acute LPS or IL-6 exposure reduced STAT3α phosphorylation in stromal cells

**Figure 1:**
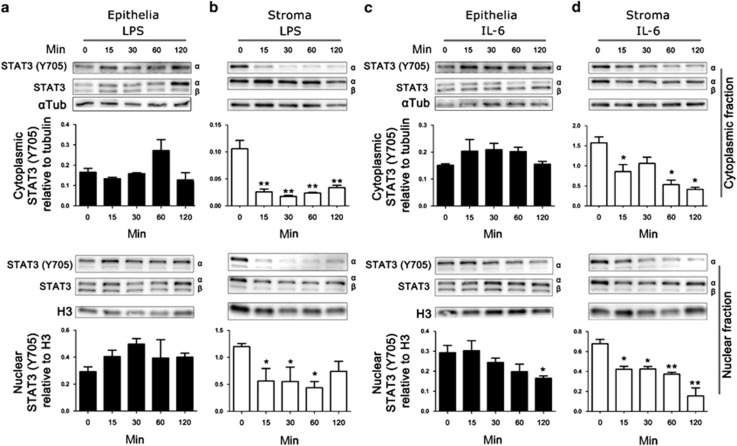
Treatment of stromal cells with lipopolysaccharide (LPS) or interleukin 6 (IL-6) rapidly reduces tyrosine phosphorylated signal transducer and activator of transcription-3 (STAT3). Epithelial (**a**, **c**) and stromal (**b**, **d**) cells were treated with LPS (**a**, **b**; 1 μg ml^−1^) or IL-6 (**c**, **d**; 10 ng ml^−1^) for 0–120 min. Cytoplasmic and nuclear cell fraction proteins were extracted, and analyzed by immunoblotting for tyrosine phosphorylated STAT3α (Y705) and total STAT3, with α-tubulin cytoplasmic or H3 nuclear controls. Average peak densities of phosphorylated STAT3 (Y705) normalized to total STAT3 are presented as mean+s.e.m. Immunoblots are representative of two experiments and analyzed by analysis of variance, using the Dunnett's multiple comparison test to compare with time 0, **P<0.01, *P<0.05.

To investigate cross-talk between the TLR4 pathway and the STAT3 pathway in endometrium, primary epithelial and stromal cells were isolated and exposed to LPS for short periods of time: from 15 to 120 min. Isolated endometrial cell populations contained less than 0.8% CD45-positive cells.^2^ Two STAT3 isoforms originate from alternate splicing, STAT3α (92 kDa) and STAT3β (84 kDa). The STAT3β isoform has been described as a dominant negative regulator of STAT3α and has a critical role in the control of systemic inflammation.^20^ Exposure of epithelial cells to LPS did not alter the abundance of STAT3α or STAT3β (Figure 1a). In stromal cells, STAT3α was more evident than the negative regulator STAT3β isoform, and abundance of STAT3α was consistent throughout LPS exposure (Figure 1b). The activation of STAT3α requires tyrosine phosphorylation (Y705) for dimerization,^21^ cytoplasmic or nuclear tyrosine phosphorylated STAT3α activation was not affected by acute exposure to LPS in epithelial cells (Figure 1a). However, stromal cells exposed to LPS showed rapid loss of cytoplasmic tyrosine phosphorylated STAT3α and nuclear STAT3α within 15 min and reduced tyrosine phosphorylated STAT3α was evident for up to 120 min after LPS exposure (Figure 1b).

The IL6R is important for the coordinated transition of leukocyte recruitment during inflammation.^6^ To investigate whether endometrial cells, can respond to IL-6, through a cognate receptor, epithelial or stromal cells were exposed to recombinant IL-6 from 15 to 120 min. Tyrosine phosphorylation of STAT3α was unaffected in the cytoplasmic fraction of LPS-exposed epithelial cells, but decreased nuclear tyrosine phosphorylated STAT3α was evident (Figure 1c). Whereas IL-6-exposed stromal cells showed a loss of cytoplasmic and nuclear tyrosine phosphorylated STAT3α within 15 min and showed reduced tyrosine phosphorylated STAT3α for up to 120 min (Figure 1d). Together, these data provide evidence for a functional IL6R on endometrial cells, and cross-talk between the TLR4 signaling pathway and the STAT3 signaling pathways in stromal cells but not epithelial cells.

### IL6R, STAT3, and SOCS3 are essential for LPS-induced IL-6 production in stromal cells but only IL6R is required in epithelial cells

**Figure 2:**
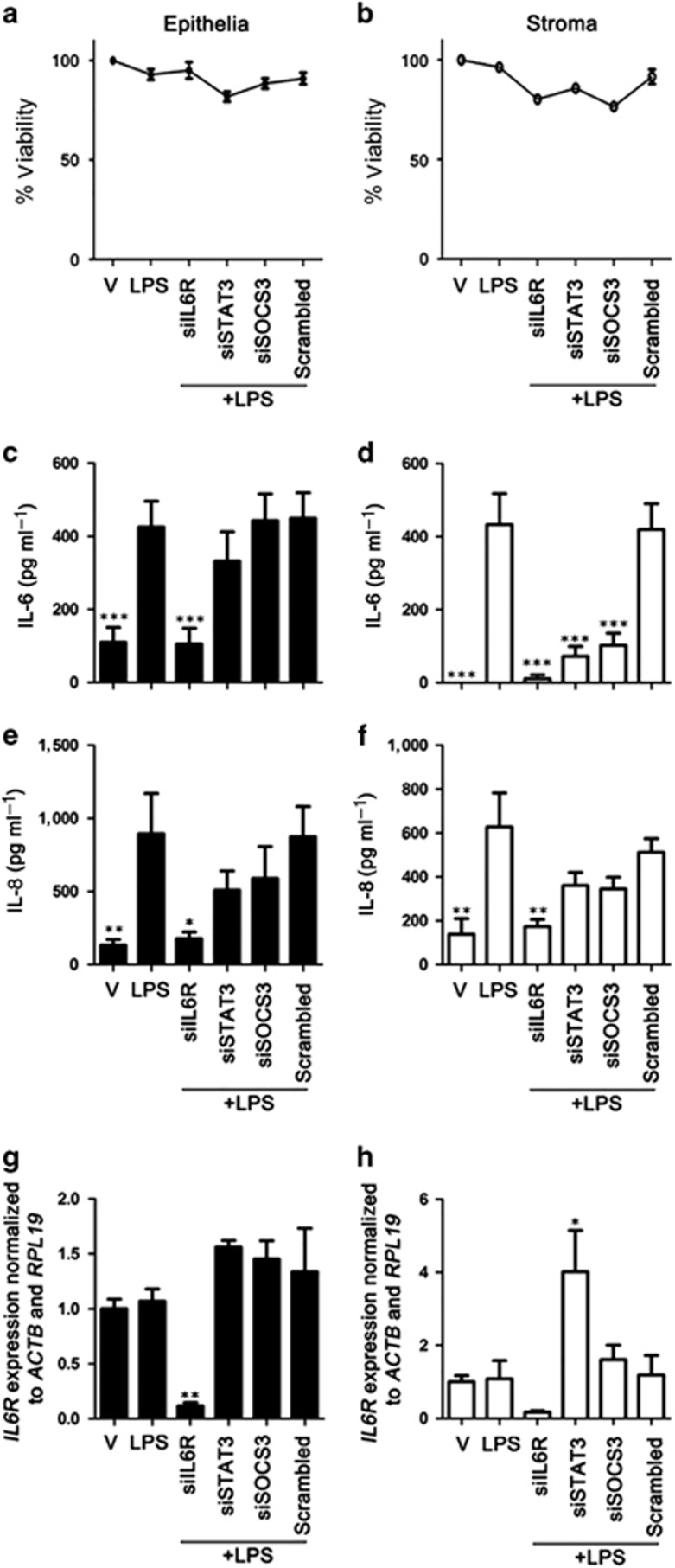
Inflammatory mediator secretion is dependent on the interleukin-6 receptor (IL6R) signaling pathway in endometrial cells. Epithelial (**a**, **c**, **e**, **g**) and stromal (**b**, **d**, **f**, **h**) cells were cultured for 24 h in medium plus vehicle (V) or media containing LPS (1 μg ml^−1^). In each independent set of experiments, cells received vehicle alone, vehicle plus short interfering RNA (siRNA) targeting *IL6R* (siIL6R), *STAT3* (siSTAT3), *SOCS3* (siSOCS3), or vehicle plus scrambled siRNA control (Scrambled) 18 h before lipopolysaccharide (LPS) treatment. Cell viability was assessed by MTT assay (**a**, **b**). Concentrations of IL-6 (**c**, **d**) and IL-8 (**e**, **f**) in cell supernatants were measured by ELISA. Data are presented as mean+s.e.m., and analyzed by analysis of variance (ANOVA), using the Bonferroni *post hoc* multiple comparison test to compare scrambled siRNA plus LPS to targeted siRNA plus LPS, ***P<0.001, **P<0.01, *P<0.05. Total RNA was extracted and the mRNA levels of *IL6R* (**g**,**h**) in cells was assessed by quantitative PCR (normalized to *ACTB* and *RPL19*). Data are presented as mean+s.e.m., and analyzed by ANOVA, using the Bonferroni *post hoc* multiple comparison test to compare scrambled siRNA plus LPS to targeted siRNA plus LPS, **P<0.01, *P<0.05. Data represent at least three independent experiments.

As endometrial stromal cells responded to exogenous IL-6, evident by rapid reduction in tyrosine phosphorylated STAT3α (Figure 1d), we hypothesized that the IL-6 produced from LPS exposed endometrial cells^1, 2^ may signal via the IL6R in a positive feedback system. To explore this, major components of the IL6R signaling pathway were targeted with short interfering RNA (siRNA). Knockdown of *IL6R*, *STAT3,* or *SOCS3* did not affect the cell viability of epithelial or stromal cells (Figure 2a and b). During 24 h LPS exposure, knockdown of *IL6R* reduced IL-6 and IL-8 accumulation in epithelial and stromal cell supernatants (Figure 2c–f). This indicates that positive feedback through the IL6R complex is required for sustained IL-6 and IL-8 production during TLR4 signaling in endometrial cells. Depletion of *STAT3* or *SOCS3* had no effect on IL-6 production in epithelial cells (Figure 2c), but stromal cells required *STAT3* and *SOCS3* for IL-6 production (Figure 2d). Furthermore, depletion of *STAT3* or *SOCS3* had no effect on *IL6R* gene expression in epithelial cells (Figure 2g), but in stromal cells knockdown of *STAT3* resulted in increased expression of *IL6R* (Figure 2h). This indicates that STAT3 has a role in limiting IL6R signaling in stroma, potentially through suppression of *IL6R* gene expression.

### IL6R and STAT3 are essential for SOCS3 mRNA expression

**Figure 3:**
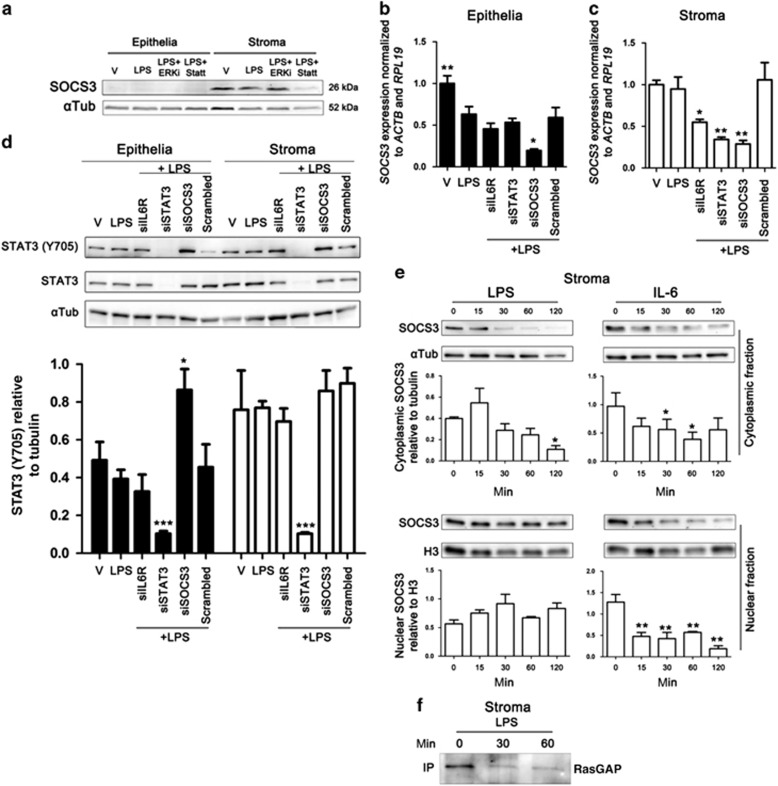
Treatment of stromal cells with lipopolysaccharide (LPS) or interleukin 6 (IL-6) reduces suppressor of cytokine signaling 3 (SOCS3) protein. Epithelial or stromal cells (**a**) were treated with vehicle, ERK inhibitor (10 μM; ERKi) or Stattic (2 μM; Statt) for 30 min before and during treatment with LPS (1 μg ml^−1^) for 24 h. Whole-cell protein was extracted and analyzed by immunoblotting for SOCS3, and α-tubulin as control. Epithelial (**b**) and stromal (**c**) cells were cultured for 24 h in medium plus vehicle (V) or media containing LPS (1 μg ml^−1^). In each independent set of experiments, cells received vehicle alone (V), vehicle plus short interfering RNA (siRNA) targeting *IL6R* (siIL6R), *STAT3* (siSTAT3), *SOCS3* (siSOCS3), or vehicle plus scrambled siRNA control (Scrambled) 18 h before LPS exposure. Total RNA was extracted and the mRNA levels of *SOCS3* were assessed by quantitative PCR (normalized to *ACTB* and *RPL19*). Data are presented as mean+s.e.m., and analyzed by analysis of variance (ANOVA), using the Bonferroni *post hoc* multiple comparison test to compare scrambled siRNA plus LPS with targeted siRNA plus LPS, **P<0.01, *P<0.05. Data represent at least three independent experiments. Endometrial cells (**d**) were cultured for 24 h in medium plus vehicle (V) or media containing LPS (1 μg ml^−1^). In each independent set of experiments, cells received vehicle alone (V), vehicle plus siRNA targeting *IL6R* (siIL6R), *STAT3* (siSTAT3), *SOCS3* (siSOCS3), or vehicle plus scrambled siRNA control (Scrambled) 18 h before LPS exposure. Whole-cell protein was extracted and analyzed by immunoblotting for STAT3 (Y705), STAT3, and α-tubulin as control. Average peak densities of STAT3 (Y705) normalized to α-tubulin are presented as mean+s.e.m. and analyzed by ANOVA, using Dunnett's multiple comparison test to compare with scrambled, ***P<0.001. Stromal cells (**e**) were treated with LPS (1 μg ml^−1^) or IL-6 (10 ng ml^−1^) for 0–120 min. Cytoplasmic and nuclear cell fraction proteins were extracted, and analyzed by immunoblotting for SOCS3 and α-tubulin cytoplasmic or H3 nuclear controls. Average peak densities of SOCS3 normalized to α-tubulin or H3 are presented as mean+s.e.m. and analyzed by ANOVA, using Dunnett's multiple comparison test to compare with time 0, **P<0.01, *P<0.05. Stromal cells (**f**) were treated with LPS (1 μg ml^−1^) for 0, 30, or 60 min. Immunoprecipitated (IP) protein was analyzed by immunoblotting using RasGAP antibody. The Immunoblot is representative of two experiments.

The SOCS family of proteins have a role in modulating TLR signaling and cytokine responses, and *SOCS3* expression was necessary for the pro-inflammatory effects of IL-6 in mouse macrophages.^15^ To explore why SOCS3 is important for LPS-induced IL-6 production in endometrial stromal cells but not epithelial cells, we next investigated SOCS3 status during TLR4 activation. Analysis of whole-cell protein by immunoblotting indicated low basal SOCS3 protein levels in untreated and LPS-exposed epithelial cells (Figure 3a). In contrast, SOCS3 protein was evident in untreated and LPS-exposed stromal cells (Figure 3a). Furthermore, SOCS3 protein was dependent on STAT3 activation shown by the reduction of SOCS3 protein in the presence of Stattic (2 μM Figure 3a), a small molecule that inhibits STAT3 activation and dimerization by binding to an SH2 domain.^22^ The transcript stability of *SOCS3* is dependent on ERK1/2 in murine fibroblasts and hepatocytes.^23^ However, an ERK1/2 activation inhibitor (10 μM), a cell-permeable peptide corresponding to the N-terminus of MEK1, had no effect on SOCS3 protein in endometrial stromal cells (Figure 3a).

We next monitored the expression of *SOCS3* mRNA after siRNA-targeted knockdown of *IL6R*, *STAT3,* or *SOCS3.* In support of the protein data, depletion of *STAT3* reduced *SOCS3* gene expression in stromal cells, but had no effect in epithelial cells (Figure 3b and c). However, despite the low abundance of SOCS3 protein in epithelial cells (Figure 3a), epithelial and stromal cells had similar levels of *SOCS3* gene expression. This low abundance of protein may be due to the stability of *SOCS3* mRNA and the short half-life of SOCS3 protein in epithelial cells.^23^ Furthermore, siRNA targeting *IL6R* reduced *SOCS3* expression in stromal cells (Figure 3c). These data indicate that *SOCS3* expression is dependent on IL6R complex signaling and STAT3 in stromal cells, providing further evidence for a positive feedback loop via the IL6R complex.

Usually, SOCS3 perturbs STAT3 dimerization and phosphorylation by blocking SH2 docking of JAK proteins.^10, 11^ Thus, we assessed whether SOCS3 had a role in blocking the tyrosine phosphorylation of STAT3α in endometrial cells. Depletion of *SOCS3* increased the tyrosine phosphorylation of STAT3α in epithelial cells but not stromal cells (Figure 3d), indicating differential roles of SOCS3 in each cell type. Interestingly, depletion of *IL6R* did not affect tyrosine phosphorylation of STAT3 (Figure 3d).

As the depletion of *SOCS3* results in reduced IL-6 production in stromal cells (Figure 2d), we investigated whether exposure to LPS or IL-6 would directly affect SOCS3 protein abundance. To this end, stromal cells were exposed to LPS or IL-6 from 15 to 120 min, and cytoplasmic and nuclear protein fractions were isolated and analyzed by immunoblotting. Here, SOCS3 was shown to reside in the cytoplasm and nuclear compartments in untreated stromal cells. However, exposure to LPS resulted in rapid loss of SOCS3 protein from the cytoplasm within 30 min, whereas nuclear SOCS3 was unaffected (Figure 3e). Exposure of stromal cells to IL-6 resulted in the loss of SOCS3 protein from both the cytoplasmic and nuclear compartment (Figure 3e). This is further evidence that SOCS3 may modulate TLR4 signaling in stromal cells.

SOCS3 is tyrosine phosphorylated in response to many growth factors and upon phosphorylation interacts with the Ras inhibitor p120 (RasGAP), resulting in sustained Ras-dependent activation of ERK1/2.^16^ Considering ERK1/2 may be important for SOCS3 stability, we next explored whether SOCS3 interacted with RasGAP using co-immunoprecipitation and immunoblot analysis. Here, endometrial stromal cells showed reduced RasGAP protein after LPS exposure, as evidenced by reduced immunoprecipitation of RasGAP protein (Figure 3f). However, no evidence of SOCS3 protein was observed in the coimmunoprecipitate (data not shown).

### Inhibition of Src kinases reduced IL-6 and IL-8 production in LPS-exposed stromal cells

**Figure 4:**
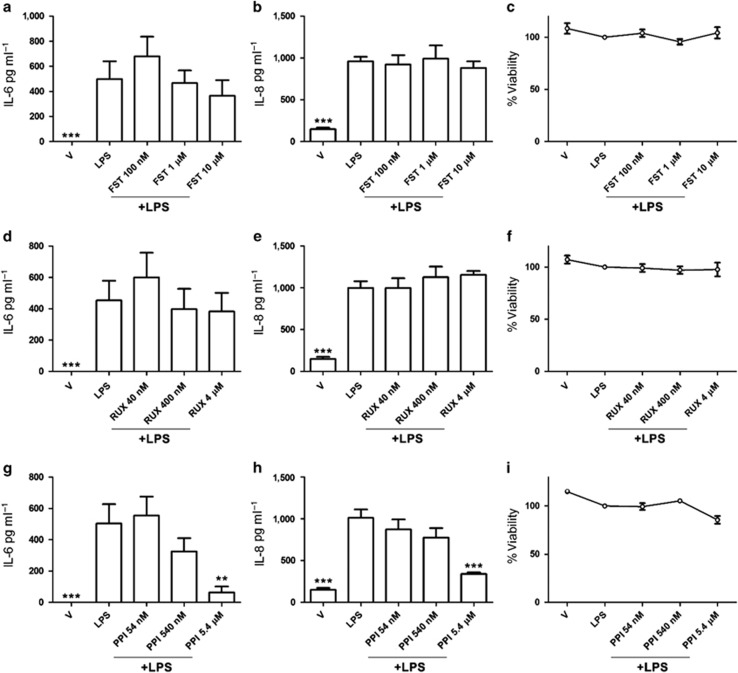
Src kinase inhibition attenuated lipopolysaccharide (LPS)-induced secretion of inflammatory mediators in stromal cell supernatants. Stromal cells (**a**–**i**) were cultured for 30 min in medium containing vehicle (V) or medium containing inhibitors (farnesyl thiosalicylic acid (FST), 100 nM, 1 μM or 10 μM; ruxolitinib (RUX), 40 nM, 400 nM or 4 μM; 1-(1,1-dimethylethyl)-3-(4-methylphenyl)-1H-pyrazolo[3,4-d]pyrimidin-4-amine (PP1), 54 nM, 540 nM or 5.4 μM) before and during treatment with control medium or LPS (1 μg ml^−1^) for 24 h. Concentrations of accumulated IL-6 (**a**, **d**, **g**) and IL-8 (**b**, **e**, **h**) in cell supernatants were measured by ELISA. Data are presented as mean+s.e.m., and analyzed by one-way analysis of variance (ANOVA), using the Bonferroni *post hoc* multiple comparison test to compare vehicle plus LPS to vehicle or vehicle plus LPS and inhibitor, ***P<0.001, **P<0.01. Stromal cell viability was assessed by MTT assay (**c**, **f**, **i**), expressed as viability compared with medium plus LPS. Data are presented as mean+s.e.m., and analyzed by ANOVA, using the Bonferroni *post hoc* multiple comparison test to compare with LPS alone.

The observed reduction in RasGAP protein (Figure 3f) did not appear to be associated with SOCS3 protein interaction, as demonstrated by coimmunoprecipitation, at the time points analyzed (30 and 60 min). Furthermore, farnesyl thiosalicylic acid, a synthetic farnesyl cysteine mimic that blocks anchorage of Ras oncoproteins to the cell membrane and to Ras-escort chaperones, had no effect on LPS-induced IL-6 or IL-8 production, or cell viability in stromal cells (Figure 4a–c). So, to explore other potential routes of cross-talk between SOCS3 and TLR4, we next investigated the role of JAK kinases. SOCS3 regulates IL6R signaling by binding to JAK kinases and gp130, interfering with STAT3 activation in response to IL-6.^10, 11^ To investigate whether cross-talk between SOCS3 activation and the TLR4 signaling pathway required JAK kinases, we used Ruxolitinib, a small-molecule ATP mimetic, which is an inhibitor of both JAK1 and JAK2. However, Ruxolitinib had no effect on LPS-induced IL-6 or IL-8 production, or on the viability of stromal cells (Figure 4d–f).

Constitutively active Src kinases, in mouse embryonic fibroblasts, are involved in the phosphorylation of IL-6-induced SOCS3, and this activation is independent of JAK kinases.^24^ So, to investigate whether Src kinases contributed to SOCS3 cross-talk with the TLR4 pathway, 1-(1,1-dimethylethyl)-3-(4-methylphenyl)-1H-pyrazolo[3,4-d]pyrimidin-4-amine (PP1), a potent, reversible, ATP-competitive, and selective inhibitor of the Src family of protein tyrosine kinases, without significantly affecting JAK2, was used during LPS exposure of stromal cells. Inhibition of Src kinases in LPS-exposed stromal cells reduced IL-6 and IL-8 production without significantly affecting cell viability (Figure 4g–i). Taken together, these data provide evidence that sustained IL-6 and IL-8 production in LPS-exposed stromal cells depends on positive feedback via the IL6R and activation of Src kinases.

### Polarized epithelial cells modulate STAT3 regulation of stromal cell IL-8 production

**Figure 5:**
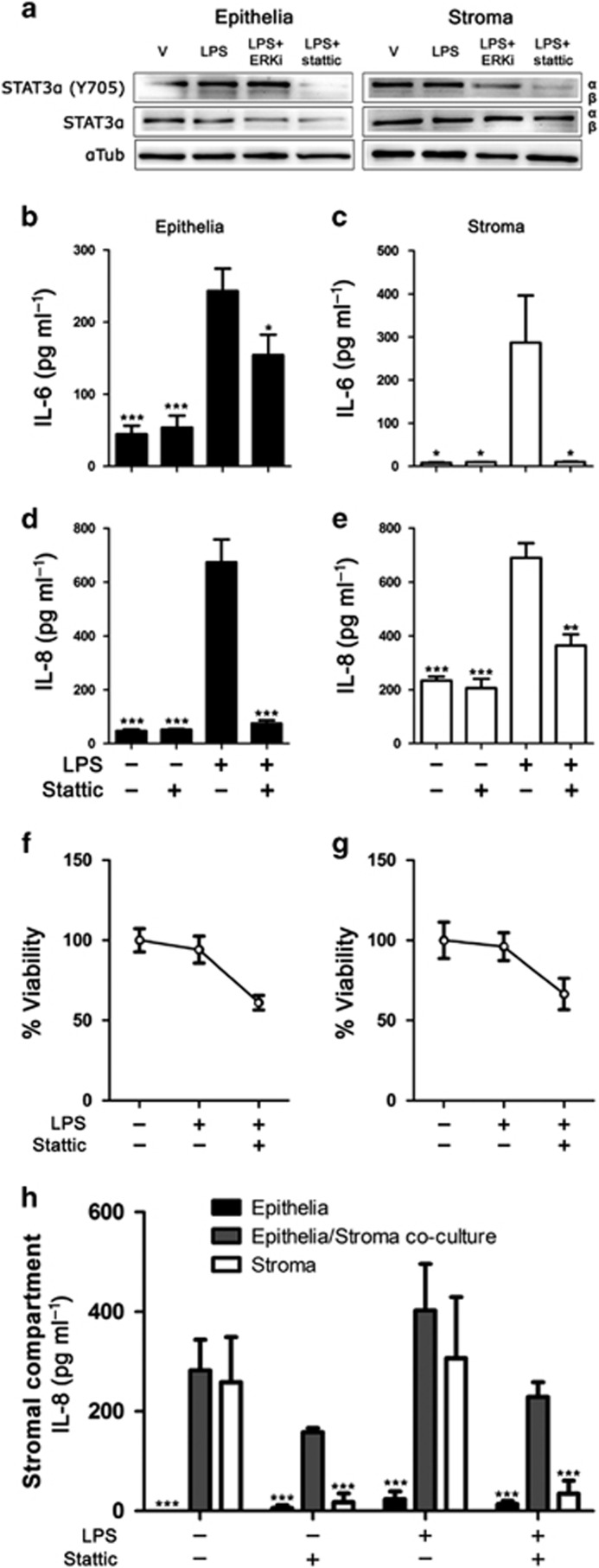
Stattic reduces inflammatory mediator secretion from endometrial cells. Epithelial or stromal cells (**a**) were treated with vehicle, ERK inhibitor (10 μM), or Stattic (2 μM) for 30 min before and during treatment with lipopolysaccharide (LPS) for 24 h. Whole-cell protein was extracted and analyzed by immunoblotting for tyrosine phosphorylated STAT3α (Y705), total STAT3, and α-tubulin as control. Epithelial (**b**, **d**, **f**) and stromal (**c**, **e**, **g**) cells were cultured for 30 min in medium containing vehicle or Stattic (2 μM) before and during treatment with control medium or LPS (1 μg ml^−1^) for 24 h. Concentrations of interleukin 6 (IL-6; **b**, **c**) and IL-8 (**d**, **e**) were measured in supernatants by ELISA. Data are presented as mean+s.e.m., and analyzed by analysis of variance (ANOVA), using the Bonferroni *post hoc* multiple comparison test to compare LPS alone with vehicle or LPS plus Stattic, ***P<0.001, **P<0.01, *P<0.05. Cell viability was assessed by MTT assay and expressed as percentage of vehicle control (**f**, **g**). Endometrial cells (**h**) were cultured as polarized epithelial monocultures in Transwell inserts, co-cultures of polarized epithelia above stromal cells, or stromal cell monocultures. The stromal compartment was treated with vehicle or Stattic (2 μM) for 30 min before and during exposure to LPS for 24 h. Concentrations of accumulated IL-8 in the stromal compartment were measured by ELISA. Data are presented as mean+s.e.m., and analyzed by ANOVA, using the Bonferroni *post hoc* multiple comparison test to compare LPS alone with vehicle or LPS plus Stattic in each treatment group, ***P<0.001.

Epithelial cells may impact stromal cell function in mucosa, and human endometrial epithelial cell secretions modulate the response to TLR ligands in dendritic cells.^25^ To begin to explore whether epithelial cells might modulate stromal cell responses to LPS during STAT3 inhibition, we exploited a co-culture model using polarized epithelial cells in a Transwell insert that can be placed above stromal cells cultured in a 24-well plate.^7^ We used Stattic (2 μM) as a strategy to be able to inhibit STAT3 in epithelial and stromal cells in co-culture, as contemporaneous siRNA of epithelial and stromal cells was impractical. We first confirmed Stattic inhibited STAT3α tyrosine phosphorylation in the presence of LPS in monocultures of epithelial and stromal cells (Figure 5a), whereas LPS alone or an ERK inhibitor in the presence of LPS had no substantive effect on STAT3α abundance. Stattic also reduced the accumulation of IL-6 and IL-8 in monocultures of epithelial cells and stromal cells that were treated with LPS (Figure 5b–e), although Stattic also reduced cell viability to a similar level in epithelial and stromal cells (Figure 5f, g). As IL-6 drives IL-8 production, we then examined how stromal cell IL-8 production in response to LPS is modulated by the presence of epithelial cells during STAT3 inhibition. We compared IL-8 accumulation in the basolateral compartment amongst polarized epithelial cell monocultures, epithelial-stromal co-cultures, and stromal cell monocultures, which were exposed to LPS and/or Stattic. The accumulation of IL-8 in response to LPS in the stromal compartment of the culture system did not differ significantly between stromal cells in monoculture and stromal cells co-cultured with epithelial cells, but the expected reduction in IL-8 accumulation in cells treated with Stattic was only evident for stromal cell monocultures, and not for stromal cells co-cultured with epithelial cells (Figure 5h). These data provide evidence that the regulation of the innate immune response by STAT3 signaling in the stroma may be modulated by epithelial cells in the endometrium.

## Discussion

During inflammation, IL6R signaling increases IL-8 production, resulting in increased neutrophil chemotaxis.^6^ Bovine endometritis is an exemplar mucosal disease, characterized by sustained neutrophil infiltration and elevated IL-6 and IL-8 during Gram-negative bacterial infection. Here, we provide evidence that STAT3 licenses TLR4-dependent IL-6 and IL-8 production via IL6R-positive feedback in endometrial cells.

**Figure 6:**
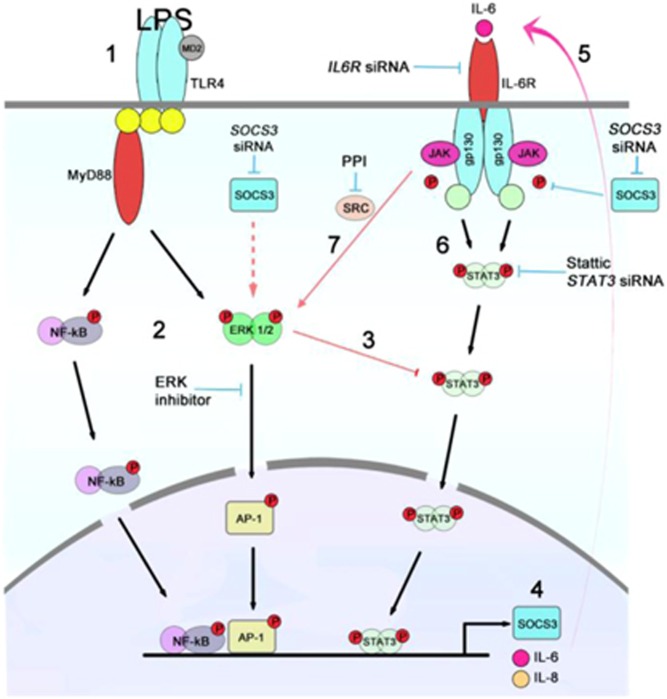
Cross-talk between interleukin 6 receptor (IL6R) and Toll-like receptor 4 (TLR4) signaling pathways in endometrial stromal cells during acute and chronic exposure to lipopolysaccharide (LPS). Acute exposure to LPS (1) activates nuclear factor-κB (NF-κB) and mitogen-activated protein kinase (MAPK) signaling pathways (2) and blocks STAT3α tyrosine phosphorylation (3) leading to the enhanced production of interkleukin (IL)-6 and IL-8 (4). IL-6 (5), through a positive feedforward loop via IL6R (6), results in tyrosine phosphorylation of STAT3α and sustained production of IL-6 and IL-8, putatively through activated Src kinase and ERK1/2 MAPK signaling pathways (7). The production of IL-6 and IL-8 is abrogated by inhibition of STAT3 or Src kinases, or by depletion of *IL6R*, *STAT3,* or *SOCS3* with short interfering RNA (siRNA; red arrows indicate putative routes of cross-talk).

To demonstrate a direct interaction between the TLR4 and STAT3 pathways, we exposed endometrial cells to LPS for short periods of time. This acute TLR4 signaling reduced tyrosine-phosphorylated STAT3 in stromal cells but did not affect STAT3 in epithelial cells. Furthermore, chronic exposure to LPS returned STAT3 to its previous tyrosine-phosphorylated state, suggesting a positive feedback system, putatively through the IL6R. Using targeted siRNA, *IL6R* proved to be critical in maintaining IL-6 and IL-8 production in epithelial and stromal cells. However, epithelial and stromal cells showed a differential signaling response in the presence of LPS when *STAT3* or *SOCS3* were targeted with siRNA. Reducing *STAT3* or *SOCS3* in epithelial cells, did not affect IL-6 or IL-8 production, whereas *STAT3* and *SOCS3* were essential for IL-6 production in stromal cells. This is in contrast to macrophages where SOCS3 is a negative regulator of IL-6 signaling.^26^ Interestingly, SOCS3 protein could not be detected in epithelial cells, but the expression levels of *SOCS3* were similar to stroma. The underlying explanation is not known, but SOCS3 protein has a short half-life and the MEK-ERK1/2 cascade is important in the regulation of *SOCS3* mRNA stability;^23^ and we have previously shown that MEK-ERK1/2 inhibition is effective at reducing IL-6 and IL-8 production in stromal cells, but not epithelial cells.^1, 2^ This suggests that ERK1/2 is important for IL-6 and IL-8 production in stromal cells through stabilization of *SOCS3* mRNA. We also considered that although the usual role of SOCS3 is to inhibit STAT3 activation, SOCS3 also binds to RasGAP, activating Ras and maintaining ERK activation (Figure 6).^16^ Here, RasGAP protein was reduced after 30 min exposure to LPS, but an association between SOCS3 and RasGAP could not be shown, and farnesyl thiosalicylic acid, an inhibitor of Ras, had no effect on IL-6 or IL-8 production.

Another potential route of cross-talk between the IL6R and TLR4 pathway is the JAK kinases. Upon ligand binding, IL6R heterodimerizes with gp130 receptor, activating JAK1, JAK2, and Tyk2 and in turn STAT3.^8, 9^ However, using the JAK 1/2 inhibitor Ruxolitinib had no effect on IL-6 or IL-8 production in endometrial stromal cells. A further consideration was that constitutively active Src kinases can interact with components of the IL6R/STAT3 pathway independently of JAK kinases.^24^ Therefore, we used an inhibitor of Src kinases, PP1, which reduced IL-6 and IL-8 production in stromal cells exposed to LPS. The differential cytokine and chemokine responses between epithelial and stromal cells were dependent on cross-talk between the IL6R and TLR signaling pathways, and involved the Src kinases, SOCS3, and STAT3 in stromal cells. Differential regulation of TLR signaling pathways via STAT3, SOCS3, and Src kinases may reflect evolutionary divergence between epidermis and mesenchyme. During parturition, the endometrium experiences extensive tissue damage, resulting in loss of epithelium, and ubiquitous contamination with bacteria. The epithelium is the first line of defense against bacteria and under normal function forms an impervious barrier; however, direct exposure of the underlying stroma to bacteria and their ligands may initiate a chain of events in stroma resulting in sustained IL-8 production and neutrophil infiltration. Excessive neutrophil infiltrate would be detrimental to the resolution of inflammation, as IL6R is shed from apoptotic neutrophils, promoting IL6R signaling. This is evidenced by neutrophil depletion resulting in reduced local soluble IL6R levels and the resolution of inflammation.^27^

The activation of IL6R leads to changes in target gene expression via the transcription factor STAT3.^4^ Signaling via IL6R and STAT3 activate factors associated with IL-8 production, including NF-κB, a transcription factor essential for *IL8* gene transcription.^28, 29^ Furthermore, in some instances, the activation of NF-κB is inhibitory to IL-6 signaling, and some genes have overlapping competitive binding sites for STAT3 and NF-κB.^30^ Temporal binding of transcription factors to gene promoters may explain why acute TLR signaling initially suppresses tyrosine phosphorylation of STAT3 in stromal cells to prevent competitive binding of STAT3 and NF-κB on the *IL6* promoter. However, chronic TLR4 signaling requires STAT3 tyrosine activation to induce cooperation between STAT3 and NF-κB on the *IL8* promoter.^29^ Indeed, in response to IL-1 receptor activation, a similar pathway to TLR4 signaling, STAT3 forms a complex with the p65 subunit of NF-κB, binding to the human *IL8* promoter, inducing gene expression.^28, 29^ This IL-1 receptor activation is in contrast to IL-6 signaling in which phosphorylated STAT3 is recruited and p65 is not.^29^ Other forms of cross-talk between the TLR and IL6R signaling pathways include the NF-κB-dependent gene expression of inhibitors of STAT3 activation, such as SOCS3 and factors that are involved in TLR signaling, including LPS-binding protein.^31^

The transition from neutrophil to mononuclear cell infiltration is an important process during resolution of mucosal inflammation.^5, 6^ However, during bovine endometritis this transition is dysregulated with sustained influx of neutrophils.^19^ Here, we show that cross-talk between TLR signaling and STAT3 pathways, in bovine endometrial cells, sustains production of IL-6 and IL-8 via IL6R signaling (Figure 6). IL-6 has been shown to amplify TLR-mediated cytokine and chemokine production in other diseases, including rheumatic inflammatory diseases.^4, 32^ Furthermore, activation of the IL6R signaling pathway in the endometrium during infection may also have consequences for fertility. For example, STAT3 and SOCS3 have critical roles in implantation and decidualization.^33, 34, 35, 36, 37^ Conversely, sustained STAT signaling results in failure of uterine implantation in mice.^38^ In humans, cAMP stimulated *SOCS3* expression during decidualization of endometrial stromal cells, but overexpression of *SOCS3* retarded decidualization.^35^ In cattle, *SOCS3* expression increases during implantation, around day 20 of pregnancy, and is induced by interferon-τ, an important signaling cytokine during embryo recognition.^39, 40^ Therefore, balanced activation of the STAT/SOCS signaling pathway is essential for fertility. However, during uterine infection, TLR4 cross-talk with the IL6R pathway may perturb this balance and these concepts need to be explored further.

The bovine endometrium readily yields purified populations of stromal and epithelial cells that are almost devoid of immune cells, which generates a tractable model for *in vitro* analysis of mucosal cells.^2, 41^ These stromal cell and epithelial cells are clearly distinct populations, as evidenced by morphology, fluorescence-activated cell sorting (FACS) analysis, and differential secretion of prostaglandins.^2, 42^ The role of STAT3 signaling in stromal but not epithelial cell innate immunity, in the present study, is further evidence that the epithelial and stromal populations are distinct. This dependence on STAT3 in stromal cells may reflect the cellular lineage, with STAT3 often considered a mesenchymal cell marker.^43^ However, there is likely heterogeneity within both the stromal and epithelial cell populations as both populations contain stem cells that can differentiate into varied tissue cells.^43^ Future work might explore if the roles of IL6R, STAT3, and SOCS3 are a consistent feature among all stromal cells, or associated with specific subpopulations of stromal cells. Although the *in vitro* cell preparations used in the present study contain very few immune cells, *in vivo* immune cells in the endometrium likely contribute to regulating the mucosal inflammatory response.^25^ A further consideration of intercellular communication is the cross-talk between stromal and epithelial cells *in vivo* and one of the features of the regulation of IL6R signaling that warrants further investigation is the relationship between epithelial and stromal cells. In the present study, there was evidence that the STAT3 regulated inflammatory response by stromal cells was modulated by the presence of epithelial cells when in co-culture. However, recent evidence shows that the inhibition of STAT3 with Stattic is less specific than first thought and has a similar binding affinity for STATs other than STAT3.^22, 44^ Future work will be challenging in the primary cells used in the present study, but insights could be obtained using conditional knock-outs in mice. The role of IL6R and STAT3 in driving inflammation may provide an opportunity for therapeutics to restrain the persistent neutrophil influx in the mucosa, even after bacteria are eliminated.

In summary, we show that STAT3 licenses TLR4-dependent IL-6 and IL-8 production in endometrial cells via the IL6R during the innate immune response. Surprisingly, there was a differential endometrial cell response, as the accumulation of IL-6 and IL-8 was dependent on SOCS3 and Src kinase signaling in stromal cells, but not epithelial cells. These findings are important because they uncover a role for the IL6R and STAT3 pathway in mucosal innate immunity, which contrast with those reported in hematopoietic cells. Furthermore, these findings could be translated to use of inhibitors of STAT3/IL6R signaling in the uterus to limit the severity of endometritis.

## Methods

**Endometrial cell culture.** Healthy uteri were collected from cattle processed as part of the normal work of an abattoir, as described previously.^1, 2^ Briefly, the endometrium was dissected from the uterus and enzymatically digested, and the endometrial epithelial and stromal cell populations were isolated by their differential adhesion to cell culture flasks. The absence of immune cell contamination was confirmed by FACS analysis, as described previously.^2^ Briefly, 1 × 10^7^ cells per ml endometrial epithelial cells, stromal cells, and whole blood were suspended in bovine serum albumin stain buffer (BD Biosciences, East Rutherford, NJ) for FACS analysis. Cells were stained with R-Phycoerythrin-conjugated mouse anti-CD45 hematopoietic cell marker (MA1–81458; Thermo Fisher Scientific, Waltham, MA); allophycocyanin-conjugated mouse anti-pan-cytokeratin (ab106166; Abcam, Cambridge, UK) as a marker of epithelia, fluorescein isothiocyanate-conjugated mouse anti-vimentin (BM5501F; Acris Antibodies, Herford, Germany) as a mesenchymal cell marker, or isotype controls, R-Phycoerythrin-conjugated anti-mouse IgG1 (PA5–33180; Thermo Fisher Scientific); allophycocyanin-conjugated anti-mouse IgG1 (406609; BioLegend Inc., San Diego, CA); or fluorescein isothiocyanate-conjugated anti-mouse IgG2a (407105; BioLegend Inc.), respectively. Analysis was performed on a BD FACSAria III cell sorter instrument using BD FACSDiva v6.1 software (BD Biosciences), using at least 1 × 10^4^ live cells.

Endometrial cells were maintained in RPMI 1640 medium, supplemented with 10% fetal bovine serum, 50 IU ml^−1^ penicillin, 50 μg ml^−1^ streptomycin, and 2.5 μg ml^−1^ amphotericin B (Sigma, St Louis, MO). However, treatments were carried out in serum-free OPTI-MEM (Invitrogen, Thermo Fisher Scientific, Waltham, MA) to exclude the possibility of soluble IL6R signaling. Cells were plated at a density of 1 × 10^5^ cells per ml in 6-, 12-, 24-well plates (Helena Bioscience, Gateshead, UK) or in 25, 75, or 150 cm^2^ flasks (Nunc, Rochester, NY).

**Endometrial cell culture treatments.** Ninety per cent confluent endometrial stromal and epithelial cells were exposed to 1 μg ml^−1^ ultrapure LPS from *E. coli* 0111:B4 (InvivoGen, Toulouse, France) or 10 ng ml^−1^ recombinant bovine IL-6 (Kingfisher Biotech, St Paul, MN). This IL-6 concentration activates STAT3 in human dendritic cells.^45^ Furthermore, IL-6 concentrations average 20 ng g^−1^ of vaginal mucus in the first week postpartum, and supernatants from endometrial explants exposed to LPS accumulate 6 ng ml^−1^ IL-6 after 24 h.^7, 46^ Each experiment used at least three animals, and treatments were replicated at least twice. Cells were collected in PhosphoSafe Extraction Reagent (EMD Millipore, Watford, UK) and stored at −20°C for immunoblotting.

**Enzyme linked immunosorbent assay.** Concentrations of bovine IL-6 were measured in supernatants by ELISA according to the manufacturer's instructions; Bovine IL-6 Screening Set (ESS0029; Thermo fisher scientific, Cramlington, UK). Bovine IL-8 was measured by ELISA as described previously.^47^

**Immunoblotting.** Cell lysate proteins were normalized to 1 μg μl^−1^ using the DC Assay (Bio-Rad, Hemel Hempstead, UK) and separated (10 μg per lane) using 10% (vol/vol) SDS-polyacrylamide gel electrophoresis, with molecular weight markers in parallel lanes (Bio-Rad). After electrophoresis, proteins were transferred to a polyvinylidene difloride membrane (Bio-Rad); nonspecific binding was blocked using 5% (wt/vol) bovine serum albumin (Sigma) in Tris-buffered saline (Sigma) for 1 h at 37 °C. Membranes were probed with antibodies targeting phosphorylated STAT3 (Y705) (#9145, Cell Signaling, Danvers, MA), STAT3 (#9139, Cell Signaling), or SOCS3 (#2932, Cell Signaling). Protein loading was evaluated and normalized by examining α-tubulin levels using α-tubulin antibody (#2144; Cell Signaling). Primary antibodies were used at 1:1,000 dilutions in Tris-buffered saline, 0.1% Tween 20 (pH 7.6; Sigma) overnight at 4°C. Membranes were washed and incubated in secondary horseradish peroxidase-conjugated antibody (Cell Signaling) in 5% (wt/vol) bovine serum albumin in Tris-buffered saline for 1.5 h, and then washed. Steady-state levels of immunoreactive proteins were visualized using enhanced chemiluminescence (Western C, Bio-Rad), and densitometry on nonsaturated immunoblots was measured using the ChemiDoc System and Quantity-one software (Bio-Rad).

**Nuclear and cytoplasmic protein extraction.** Nuclear and cytoplasmic proteins were extracted according to the manufacturer's instructions (Active Motif Europe, Rixensart, Belgium). Briefly, cells were grown to ∼70% confluency, treated, and collected in ice-cold PBS containing phosphatase inhibitors, and hypotonic buffer used to make the cell membrane fragile. A detergent allowed leakage of cytoplasmic proteins and their collection in supernatant. Nuclei were lysed and nuclear proteins solubilized in lysis buffer containing protease inhibitors. Nuclear and cytoplasmic proteins were separated (5 μg per lane) using 10% (vol/vol) SDS-polyacrylamide gel electrophoresis, and immunoblotted as before, using α-tubulin and H3 (ab1791, Abcam) to assess cytoplasmic and nuclear fraction purity, respectively.

**Short interfering RNA.** Endometrial epithelial and stromal cells were transfected with Lipofectamine RNAiMAX Reagent (Invitrogen, Waltham, MA) and siRNA (designed using Dharmacon's siDESIGN Center, gelifesciences.com) targeting *IL6R*, *STAT3*, or *SOCS3* (duplex sequences in Supplementary Table 1 online). Briefly, RNAiMAX–RNAi duplex complexes were formed by adding 50 pmol of siRNA to 500 μl of Opti-MEM I medium (11058-021, Invitrogen) in each well of a six-well plate, with 50 pmol of ON-TARGETplus Non-targeting siRNA #1 (Dharmacon; gelifesciences.com) as a control. Then, 7.5 μl RNAiMAX was added to each well containing the diluted RNAi molecules and incubated for 20 min at room temperature. Exponentially growing cells (7 × 10^5^ epithelial cells, 5 × 10^5^ stromal cells) were then seeded in 2.5 ml per well of RPMI 1640 growth media, supplemented with 10% fetal bovine serum. All transfections were replicated in triplicate, and LPS (1 μg ml^−1^) treatments were carried out in OPTI-MEM medium within 48 h for RNA studies, or 72 h for protein studies.

**Quantitative PCR (qPCR).** Cells were washed with PBS, and RNA extracted using the RNeasy Mini Kit and automated Qiacube system, according to the manufacturer's instructions (Qiagen, Crawley, UK). Extracted RNA was quantified (Nanodrop ND1000 spectrophotometer, Labtech, Ringmer, UK), and 1 μg of total RNA added to a genomic DNA elimination reaction, followed by conversion to cDNA (Quantitect Reverse Transcription Kit, Qiagen), according to the manufacturer's instructions. Quantitative PCR was performed using intron-spanning primers (Supplementary Table S2) and the IQ5 system (Bio-Rad, Hemel Hempstead, UK). The starting quantity of mRNA from experimental samples were determined using standard curves generated from serial dilutions of pooled reference RNA with Quantifast SYBR green (Qiagen). Sample and reference genes were analyzed in triplicate, and mRNA expression normalized to *ACTB* and *RPL19* (Supplementary Table S2) using the IQ5 system (Bio-Rad), with inter-run correlation and run-dependent differences corrected using qBase software on the IQ5 system (Bio-Rad).^48^ The *ACTB* and *RPL19* reference genes did not differ in expression with treatment, were amplified at the same efficiency as the target gene, and had target stability mean *M* values below recommended thresholds of <0.3 and <0.6 for epithelial (homogenous) and stromal cells (heterogeneous), respectively.^48^

**Co-immunoprecipitation.** Stromal cells grown to 90% confluence in 150 cm^2^ flasks were exposed to 1 μg ml^−1^ ultrapure LPS (InvivoGen) for 0, 30, or 60 min. Immunoprecipitations were carried out with 25 mg ml^−1^ total protein and 50 μg monoclonal antibody RAS P21 Protein Activator 2 (RasGAP; Abcam, ab13057) linked to 10 ng of Dynabeads (Life Technologies, Paisley, UK) and incubated for 2 h at room temperature. For immunoblot analysis, immunoprecipitated protein was detected using RasGAP antibody (Abcam, ab13057) or SOCS3 (#2932, Cell Signaling).

**Inhibitors.** Endometrial cells were incubated for 30 min before and during exposure to 1 μg ml^−1^ LPS with the following inhibitors: Stattic (6-nitrobenzo[b]thiophene-1,1-dioxide; Millipore, UK); ERK1/2 activation inhibitor peptide I (Merck, Nottingham, UK); farnesyl thiosalicylic acid (Santa Cruz Biotechnology, Inc., Heidelberg, Germany); Ruxolitinib (RUX; InvivoGen); PP1 (1-(1,1-dimethylethyl)-3-(4-methylphenyl)-1H-pyrazolo[3,4-d]pyrimidin-4-amine; Cayman Chemical, Cambridge, UK); using dimethyl sulfoxide as vehicle control. Cell viability was evaluated at 6 or 24 h by 3-(4,5-dimethylthiazol-2-yl)-2,5-diphenyltetrazolium bromide (MTT) assay.

**Co-culture.** Polarized epithelial cells were prepared by seeding 3 × 10^5^ cells on each Millicell hanging cell culture insert (Millipore, Watford, UK), pre-coated with Matrigel (BD Biosciences, Heidelberg, Germany), and placed in 24-well plates (TPP, Helena Biosciences, Gateshead, UK), with 300 and 800 μl RPMI culture medium (supplemented with 10% fetal bovine serum, 50 IU ml^−1^ of penicillin, 50 μg ml^−1^ of streptomycin and 2.5 μg ml^−1^ of amphotericin B) in the apical and basolateral compartment, respectively, as described.^49^ Epithelial layer confluence was determined by trans-epithelial resistance>1,000 Ωcm.^49^ Stromal cells were seeded (1 × 10^4^) into the basolateral compartment of the culture plates below the polarized epithelial cells or cell-free inserts 24 h before treatment. Cells were then co-cultured and treated for a further 24 h with OPTI-MEM media alone or containing LPS, with or without Stattic (2 μM) in the basolateral compartment. Experiments were repeated using three animals, and basolateral supernatants collected for ELISA.

**Statistics.** The animal was designated as the statistical unit, and data presented as mean (s.e.m.). Treatments were compared by analysis of variance (ANOVA) with Bonferroni's or Dunnett's post-comparison test, using SPSS 16.0 (SPSS, IBM Corp., Armonk, NY), with *P*<0.05 regarded as significant.

## Notes

The authors declared no conflict of interest.
