# Supplementary material for: Signal transducer and activator of transcription-3 licenses Toll-like receptor 4-dependent interleukin (IL)-6 and IL-8 production via IL-6 receptor-positive feedback in endometrial cells
Source: Mucosal Immunol. 2016 Jan 27;9(5):1125–36. doi: 10.1038/mi.2015.131 (PMC4990777; doi:10.1038/mi.2015.131)
Supplement: Supplementary Tables [file mi2015131x1.docx]

SUPPLEMENTARY MATERIAL

Supplemental Table 1: siRNA sequences and NCBI Reference Sequences.

| **Target** | **Sense** | **Antisense** | **Accession #** |
| --- | --- | --- | --- |
| IL6R | GACAACACAUCGAGGAACAUU | UGUUCCUCGAUGUGUUGUCUU | NM_001110785 |
| STAT3 | CUGAAAUAAUCAUGGGCUAUU | UAGCCCAUGAUUAUUUCAGUU | NM_001012671 |
| SOCS3 | CAACAUCUCUGUCGGAAGAUU | UCUUCCGACAGAGAUGUUGUU | NM_174466 |

Supplemental Table 2: PCR primer sequences, amplicon length and NCBI Reference Sequences.

| **Target** | **Forward** | **Reverse** | **Accession #** |
| --- | --- | --- | --- |
| IL6R | CCCCCTCATCTCCCCACCGG | GCCCCAGGCTGCTAGGCCTA | NM_001110785 |
| SOCS3 | ACAGCAAGTTTCCCGCCGCC | TCGGCGCTCAGCAGCAAGTT | NM_174466 |
| RPL19 | ATGCCAACTCCCGCCAGCAGAT | TGTTTTTCCGGCATCGAGCCCG | NM_001040516 |
| ACTB | CAGAAGGACTCGTACGTGGG | TTGGCCTTGGGGTTCAGGG | NM_173979 |
